# Dietary intake of polyunsaturated fatty acids, their food sources and fertility in females and males: a preconception prospective population-based cohort study

**DOI:** 10.1016/j.ajcnut.2025.04.006

**Published:** 2025-04-11

**Authors:** Mireille C Schipper, Vincent WV Jaddoe, Eline L Bekkers, Annemarie GMGJ Mulders, Romy Gaillard

**Affiliations:** 1 The Generation R Study Group, Erasmus MC, University Medical Center, Rotterdam, The Netherlands; 2 Department of Pediatrics, Sophia’s Children’s Hospital, Erasmus MC, University Medical Center, Rotterdam, The Netherlands; 3 Department of Obstetrics and Gynaecology, Erasmus MC, University Medical Center, Rotterdam, The Netherlands

**Keywords:** fertility, PUFA, seafood, nuts, omega-3, omega-6

## Abstract

**Background:**

Seafood, nuts, and seeds are key dietary sources of polyunsaturated fatty acids (PUFAs), which may benefit reproductive health.

**Objectives:**

This study aims to investigate associations of periconception dietary intake of PUFA-rich foods and omega-3 and omega-6 PUFAs with fecundability and subfertility in females and males.

**Methods:**

Among 830 females and 651 males, participating in a population-based prospective cohort study from preconception onwards, we assessed periconception dietary intake at median 12.4-wk gestation (95% range: 10.9, 18.4) and time to pregnancy via questionnaires. Fecundability was defined as the probability of conceiving within 1 mo and subfertility as time to pregnancy ≥12 mo or use of assisted reproductive technology. Cox proportional hazards and logistic regression were used to assess associations between PUFA(-rich foods) with fecundability and subfertility.

**Results:**

In females, PUFA-rich food intake was not significantly associated with fertility. Higher omega-3 PUFA intake, especially docosahexaenoic acid and alpha-linolenic acid, but not omega-6 PUFA, was per standard deviation score (SDS) increase associated with increased fecundability and reduced subfertility. Strongest effects were observed for females in the highest quartile. A lower omega-6 to omega-3 PUFA ratio was associated with increased fecundability and lower subfertility risk [fecundability ratio (FR): 0.92, 95% confidence interval (CI): 0.87, 0.96; odds ratio (OR): 1.14, 95% CI: 1.02, 1.26, per unit increase in omega-6:omega-3 PUFA ratio]. In males, higher intake of nuts/seeds, but not seafood, was associated with increased fecundability and lower subfertility (FR: 1.10, 95% CI: 1.01, 1.20; OR: 0.78, 95% CI: 0.63, 0.97 per SDS increase in nuts/seeds). Strongest effects were present for those who consumed the highest amount. No associations were observed for omega-3 or omega-6 PUFA intake in males.

**Conclusions:**

In females, higher dietary omega-3 PUFAs intake may benefit fertility. Increased nuts and seeds consumption may improve fertility in males, independent of PUFA intake. These findings suggest potential for gender-specific dietary interventions to support reproductive health.

## Introduction

Subfertility, defined as the inability to conceive after 12 mo of regular, unprotected intercourse, affects millions of couples globally [1,2]. Although medical treatments exist, they have modest success rates, are expensive, and create a physiological burden. Accumulating evidence suggests that diet, particularly intake of poly-unsaturated fatty acids (PUFAs), may play a role in optimizing fertility naturally [3,4].

PUFAs, including omega-3 and omega-6 fatty acids, are essential nutrients and must be obtained from diet. Global dietary guidelines recommend regular consumption of seafood, ∼2 servings per week, as a key source of omega-3 PUFAs, and daily consumption of 25–30 g nuts and seeds, as key sources of omega-6 PUFAs [5,6]. PUFAs support reproductive processes, serving as precursors for prostaglandin synthesis, which regulates ovulation, implantation, and sperm function [7]. Omega-3 PUFAs may particularly be beneficial for fertility, as they reduce inflammation and support endometrial receptivity. Although omega-6 PUFAs are thought to contribute to ovarian steroidogenesis, excessive omega-6 PUFAs may exert proinflammatory effects [7]. Spermatozoa require a high PUFA content for membrane fluidity, essential for successful fertilization [4,7]. Beyond their individual roles, the omega-6 to omega-3 PUFA ratio may also be relevant for fertility, as a lower omega-6 to omega-3 PUFA ratio has been associated with improved sperm quality and ovarian structure in rats [8,9].

Despite their biological relevance, research on dietary PUFAs and human fertility remains inconclusive. PUFA-rich foods also contain other nutrients and bioactive compounds, which may influence fertility and even dilute potential beneficial effects. Studying both PUFA-rich foods and dietary PUFA intake directly is essential for a more comprehensive understanding of their role in fertility. Among 229 couples undergoing assisted reproductive technology, higher dietary intake of omega-3 PUFAs and PUFA-rich foods, specifically seafood but not nuts, was positively associated with the probability of live birth in females [10]. In males, dietary intake of omega-3 PUFAs, but not PUFA-rich foods, was positively associated with semen quality parameters, although these associations were unrelated to assisted reproductive technology outcomes [10]. However, these findings are derived from populations undergoing infertility treatment, which limits their generalizability to couples attempting conception without medical assistance. Two web-based preconception cohort studies in Denmark and North America examined the associations between PUFA-rich foods and PUFA intake and fecundability among females attempting to conceive naturally [11]. Fecundability, an important fertility marker, reflects the probability of conceiving within 1 mo. Dietary intake of omega-3 PUFAs, but not seafood intake, was associated with higher fecundability in North-American females only [11]. No studies explored the associations between PUFA-rich foods or dietary PUFA intake and fecundability in males in the general population. Understanding independent and combined effects of PUFA-rich foods and dietary PUFA intake in both females and males from the general population on their time to pregnancy is crucial for optimizing couples’ fertility and informing comprehensive preconception care strategies.

Therefore, in this prospective population-based cohort study from preconception onward, we first aimed to investigate the associations between periconception dietary intake of PUFA-rich foods in both females and males from the general population with fecundability and subfertility risk. Second, to explore whether observed associations were related to dietary PUFA intake, we examined the associations of dietary omega-3 and omega-6 PUFA intake in females and males with these fertility outcomes directly.

## Methods

### Study population

This study was nested within the Generation R *Next* Study, a population-based prospective cohort study from the preconception period onwards in the city of Rotterdam, The Netherlands, and is part of the Generation R Study Programme [12]. The general aim of this study is to identify preconception and early-pregnancy determinants of fertility, embryonic development, and childhood outcomes. Females and their partners in the general population were eligible if they were ≥18 y old, residing in Rotterdam, and actively trying to conceive or already pregnant. Couples were included in the preconception period or pregnancy between 9 August, 2017 and 1 July, 2021. Follow-up of parents and their offspring is still ongoing and planned until young adulthood. Although enrollment aimed for inclusion during preconception or early pregnancy, enrollment was allowed until delivery. For this substudy on dietary intake, a randomly selected subgroup of females and their partners participating from preconception or early pregnancy onwards were included. Only this subgroup was invited to participate in studies on dietary intake data using a food frequency questionnaire (FFQ), based on our study design and considerations of participant burden. Of the participants in our substudy, 35.5% enrolled during the preconception period, whereas 64.5% enrolled during early pregnancy (median 10.1 wk of gestation). At the time of dietary assessment, all females were pregnant. Supplemental Figure 1 provides a timeline illustrating the study design, enrollment moments, and timing of data collection. Study approval was obtained by the Medical Ethical Committee of the Erasmus University Medical Centre, Rotterdam (MEC 2016-589, NL57828.078.16). Written informed consent was obtained from participating females and males. In total, 1054 unique females who enrolled between 2017 and 2021 had information on dietary intake. Of these, 830 unique females had information on time to pregnancy, with 651 unique females having male partners who also provided information on dietary intake. Details are present in the flowchart (Supplemental Figure 2).

### Periconception PUFA-rich food intake and dietary PUFAs in females and males

We obtained information on female and male periconception dietary intake at a median of 12.4 wk of gestation (95% range: 10.9, 18.4), using the Dutch version of the semiquantitative HEalthy LIfe in an Urban Setting (HELIUS) FFQ, as described previously [[13], [14], [15]]. In short, females and males reported the frequency of consumption of 238 food items over the past 4 wk, with options ranging from never to 7 times per week. Mean daily energy and nutrient intake was calculated by linking food items in the questionnaire to 1 or more foods from the Dutch Food Consumption Table [Netherlands Nutrition Centre (2011) NEVO: Dutch food composition database 2011, Netherlands Nutrition Center, the Hague], and subsequently multiplying the frequency of consumption by the consumed amounts and nutrient content per item. This dietary assessment served as a measure of periconception diet, as maternal dietary patterns tend to remain stable from preconception to early pregnancy [14,16,17].

Total seafood intake was calculated as the sum of lean fish, fatty fish, shellfish, and ultraprocessed fish (e.g. breaded or fried) (grams per day). We further categorized seafood intake according to consumption frequency (<1 d/wk, 1 d/wk, ≥2 d/wk). We assessed adherence to the Dutch dietary recommendation for seafood for females wishing to conceive or pregnant and for males (≥2 servings seafood per week). We classified couples as neither partner consumes ≥2 servings per week, 1 partner consumes ≥2 servings per week or both partners consume ≥2 servings per week [18,19]. Total nuts and seeds intake was calculated as the sum of nuts, seeds, and nut-based spreads (grams per day). We categorized nuts and seeds intake into 2 primary subgroups: peanut products, and other nuts and seeds (such as walnuts, almonds, and flaxseeds). This distinction may capture potential variations in nutrient profiles that could differentially influence fertility outcomes. We assessed adherence to the Dutch dietary recommendation for nuts and seeds (≥25 g of nuts and seeds daily), and classified couples as neither partner consumes ≥25 g/d, 1 partner consumes ≥25 g/d or both partners consume ≥25 g/d [20]. Dutch dietary recommendations for pregnant females and those trying to conceive closely align with those in other Western countries, such as North America, Australia, and the United Kingdom, where guidelines generally advise 2–3 servings of fish per week for females of childbearing age and those pregnant [5,21]. Similarly, international recommendations for nuts and seeds intake typically range from 20 to 30 g/d [6]. Although plant-based oils contain PUFAs, the ones consumed most within our population, olive oil and sunflower oil, provide only minimal amounts of omega-3 PUFAs. Given that our primary focus was on dietary sources rich in omega-3 PUFAs and the omega-6 to omega-3 ratio, we did not consider plant-based oils as primary exposure in our analysis.

Potential beneficial effects of PUFA-rich foods on fertility may also be influenced by other nutritional components present in these foods. To examine whether observed effects were directly related to dietary omega-3 or omega-6 PUFAs, we considered dietary intake of total omega-3 PUFAs, total omega-6 PUFAs, and the omega-6 to omega-3 ratio, as secondary exposures. We further assessed omega-3 PUFAs individually, including alpha-linolenic acid (ALA), eicosapentaenoic acid (EPA), and docosahexaenoic acid (DHA), as they serve distinct biological roles. ALA, primarily found in plant sources, acts as a precursor for EPA and DHA. EPA and DHA, found predominantly in seafood, are involved in anti-inflammatory pathways and membrane integrity. We calculated SD scores (SDS) of dietary intake of seafood, nuts/seeds, omega-3, and omega-6 PUFAs. We used the analyses per SDS to assess associations across the full range of dietary PUFA-rich foods and PUFA intake to identify small effects across the full range relevant on a population-level. We considered these our main analyses and refer to these analyses as effects across the full range. For PUFA-rich foods, we also examined associations using categories based on consumption frequency and amounts to enhance clinical applicability. Because there are no predefined dietary recommendations or intake cut-offs for PUFAs as nutrients, we additionally considered total dietary omega-3 and omega-6 PUFA intake in quartiles to identify any potential threshold effects.

### Time to pregnancy

Time to pregnancy and mode of conception were assessed through questionnaires in preconception and early pregnancy. We used questions regarding the date at which females started trying to conceive and refrained from using contraceptives (start date actively pursuing pregnancy) and the date of assisted reproductive technology, including intrauterine insemination, ovulation induction, in vitro fertilization, and intracytoplasmic sperm injection. The first day of the last menstrual period was obtained from the obstetric caregiver. Time to pregnancy was calculated from the start date of actively pursuing pregnancy and their first day of the last menstrual period. In females who used assisted reproductive technology, we added 12 mo to their time to pregnancy, as fertility treatments usually start after 1 y of not conceiving [22]. Fecundability was defined as the probability of conceiving within 1 mo. Time to pregnancy was categorized in 2 groups: <12 mo (fertile) and ≥12 mo (subfertile). Females who underwent assisted reproductive technology were included in the subfertile group.

### Covariates

Information on age (years), ethnicity (Dutch, European, non-European), and highest education level (low/normal, high) was obtained through questionnaires at enrollment [23,24]. Ethnicity was based on questions regarding the birth country of the participants and their parents and classified according to statistics Netherlands [24]. If one of the participants’ parents was born abroad, participants were classified as of non-Dutch ethnic origin; if both parents were born abroad, the birth country of the mother defined the participants’ ethnicity. Information on smoking (yes, no), alcohol use (yes, no), and drug use (yes, no) before pregnancy, folic acid supplementation use (yes, no), PUFA supplement use (yes, no), parity (nulliparous, multiparous), history of sexual transmitted disease (yes, no), and vomiting or nausea during the first trimester of pregnancy (yes, no) was assessed through questionnaires filled in during preconception and/or early pregnancy. Preconception BMI in females and BMI in males were assessed either via clinical measurements of height and weight at time of enrollment without shoes and heavy clothing, or by questionnaires. BMI was categorized into underweight/normal weight (≤24.9 kg/m^2^) and overweight/obesity (≥25 kg/m^2^). Underweight (females: *n* = 29, males: *n* = 3) and normal weight (females: *n* = 554, males: *n* = 390) categories were combined due to the small number of underweight participants.

### Statistical power

Power calculations were performed based on a sample size of 700 subjects. For a normally distributed continuous outcome, we could detect a difference of 0.35 SDs with a 5% type I error rate and 20% type II error rate (80% power), assuming that 10% of subjects are exposed to the relevant factor. For subfertility risk, with the same type I and II errors, we were able to detect an odds ratio (OR) of 2.48, given that 10% of participants have the relevant exposure of interest and the incidence of subfertility risk is 10% [25]. These differences are similar as differences that could be detected in previous studies.

### Statistical analysis

First, we performed a nonresponse analysis comparing characteristics of females and males who were eligible for the FFQ but did not respond with those who did respond using *t*-tests, Mann–Whitney *U*-tests, Chi-square tests, or Fisher’s exact tests.

Second, we examined the associations of dietary intake of seafood and nuts/seeds across the full range in SDS and in (frequency/consumption) categories with fecundability in females and males using Cox proportional hazard models (R package *survival*). The survival outcome was conception. The time variable was time to pregnancy (months/28 d). The resulting hazard ratios from the Cox proportional hazard models represent the fecundability ratios (FRs). We also examined the associations of dietary intake of seafood and nuts/seeds in SDS and in categories with the risk of subfertility in females and males using logistic regression models. For clinical translation to nutritional recommendations, we further examined the effects of adherence to Dutch dietary recommendations for seafood, nuts, and seeds consumption in females and males on fecundability and subfertility using similar models.

Third, we explored whether the associations of PUFA-rich foods with fertility outcomes were directly related to dietary PUFA intake. We examined the associations of dietary intake of total omega-3 PUFAs, total omega-6 PUFAs, omega-6 to omega-3 PUFA ratio, ALA, EPA, and DHA across the full range in SDS, with fecundability and subfertility using Cox proportional hazard models and logistic regression models, respectively. Additionally, we explored the associations of dietary intake of total omega-3 and omega-6 PUFAs in quartiles with fecundability and subfertility risk, using similar models.

We specifically aimed to identify independent and combined effects of dietary intake of PUFA-rich foods and PUFAs in females and males on fertility outcomes, and examined associations in females and males singularly and simultaneously in combined models. To estimate independent effects, we conducted separate models, including either maternal PUFA intake with female-specific covariates or paternal PUFA intake with male-specific covariates. To estimate combined effects, we ran mutually adjusted combined models that included both maternal and paternal PUFA intake, adjusting for both male and female confounders. The statistical interaction terms between dietary PUFA and PUFA-rich food intake of females and males were not statistically significant. All regression models were first analyzed in univariate models and second in multivariate models with adjustment for potential confounders. Potential confounders were selected a priori based on a directed acyclic graph and associations of the exposure and outcome in existing literature (Supplemental Figure 3) [[26], [27], [28], [29], [30]]. We adjusted models for females for age, ethnicity, educational level, alcohol use, smoking, parity, BMI, and total energy intake. The models for males were adjusted for age, ethnicity, educational level, alcohol use, smoking, BMI, and total energy intake. Combined models for both females and males included all above-mentioned covariates. We explored inclusion of more dietary factors; however, due to multicollinearity issues, these were not included in the final models.

For the Cox proportional hazard models, we checked the proportional hazard assumptions of the covariates using Schoenfeld residuals, assessed linearity of all associations using Martingale residuals, and assessed influentials using deviance residuals. Violations were detected for ALA, total omega-3 PUFAs, total omega-6 PUFAs, prepregnancy BMI, and parity in females. To address this, time-transformed covariates were included to the models [31]. We performed 2 sensitivity analyses. First, we repeated the analyses restricted to females and males with a prepregnancy BMI ≥25, as dietary components, including omega-3 and omega-6 PUFAs, may exert different effects in individuals with higher BMI due to metabolic and inflammatory changes associated with adiposity. Second, we conducted a sensitivity analysis adjusting for PUFA supplement use before pregnancy to account for potential confounding from supplemental intake.

Missing values were imputed using multiple imputation by chained equations to reduce potential bias due to missing values of covariates (R package *mice*). Pooled results were reported. The percentage of missing values for covariates ranged from 0% to 19.3%. From a hypothesis-generating perspective, we considered nominal *P* values <0.05 significant. Analyses were performed using R Statistical Software version 4.2.1 and SPSS version 28.0.1.0.

## Results

### Population characteristics

Median time to pregnancy was 4.8 mo (IQR: 1.2–16.3), with 30.5% of 830 females experiencing subfertility. Median daily seafood consumption was 15.4 g (IQR: 5.1–26.3) for females and 19.3 g (IQR: 9.6–31.5) for males, whereas median daily nuts and seeds consumption was 10.1 g (IQR: 4.1–19.4) and 14.1 g (IQR: 6.4–28.1), respectively. Both males and females consumed the highest amounts of lean and fatty fish (median 8.8 g, IQR: 0.7–17.4 in females and median 11.0 g, IQR: 2.1–18.2 in males), whereas shellfish and ultraprocessed fish were consumed in lower amounts (Table 1).

**TABLE 1:** Population characteristics.

	Females (*n* = 830)	Males (*n* = 651)
Population characteristics		
Age at dietary intake assessment, mean (SD) (y)	32.1 (3.9)	34.2 (5.0)
Gestational age at dietary intake assessment, median (95% CI) (wk)	12.4 (10.9, 18.4)	—
Ethnicity, % (*n*)		
Dutch	69.0 (570)	75.0 (486)
European	9.6 (79)	7.3 (47)
Non-European	21.4 (177)	17.7 (115)
Education level, high, % (*n*)	82.4 (674)	75.0 (487)
(Prepregnancy) BMI, median (IQR) (kg/m^2^)	23.0 (21.1–25.3)	24.6 (22.7–26.8)
Overweight/obesity, % (*n*)	27.6 (222)	44.3 (286)
Smoking before pregnancy, % (*n*)	41.7 (315)	48.4 (313)
Alcohol use before pregnancy, % (*n*)	85.2 (702)	92.3 (597)
Drug use before pregnancy, % (*n*)	8.7 (72.0)	15.3 (99)
Periconception folic acid supplement use, % (*n*)	99.5 (804)	—
PUFA supplement use before pregnancy, % (*n*)	16.9 (140)	—
Parity, nulliparous, % (*n*)	72.5 (595)	—
Previous miscarriage, % (*n*)	19.1 (142)	—
Daily vomiting and nausea during early pregnancy, % (*n*)	2.5 (20)	—
Treated for a sexually transmitted disease, % (*n*)	22.9 (170)	—
Energy intake, mean (SD) (kcal/d)	1870.8 (500.8)	2348.1 (594.1)
Carbohydrate intake, mean (SD) (g/d)	215.4 (62.1)	250.0 (69.1)
Protein intake, mean (SD) (g/d)	74.5 (22.2)	95.6 (26.5)
Fat intake, mean (SD) (g/d)	68.2 (22.5)	87.9 (28.2)
Fiber intake, mean (SD) (g/d)	23.0 (6.9)	26.1 (8.3)
Total seafood intake, median (IQR) (g/d)	15.4 (5.1–26.3)	19.3 (9.6–31.5)
Shellfish intake, median (IQR) (g/d)	0.9 (0.0–8.9)	3.6 (0.0–8.9)
Lean and fatty fish, median (IQR) (g/d)	8.8 (0.7–17.4)	11.0 (2.1–18.2)
Ultraprocessed fish, median (IQR) (g/d)	0.0 (0.0–4.3)	0.0 (0.02–4.3)
Seafood consumption frequency, % (*n*)		
<1 d/wk	49.0 (407)	42.7 (278)
1 to <2 d/wk	31.2 (259)	36.7 (239)
≥2 d/wk	19.8 (164)	20.6 (134)
Adhering to seafood recommendation,^1^ % (*n*)	19.8 (164)	20.6 (134)
Nuts and seeds intake, median (IQR) (g/d)	10.1 (4.1–19.4)	14.1 (6.4–28.1)
Nuts and seeds consumption categories, % (*n*)		
Low intake: 0–10 g	49.2 (408)	37.2 (242)
Moderate intake: >10–20 g	26.6 (221)	27.0 (176)
High intake: >20 g	24.2 (201)	35.8 (233)
Adhering to nuts and seeds recommendation,^1^ % (*n*)	16.6 (138)	28.9 (188)
Omega-3 PUFAs, mean (SD) (g/d)	1.8 (0.7)	2.2 (0.9)
ALA, mean (SD) (g/d)	1.5 (0.6)	1.8 (0.8)
EPA, mean (SD) (g/d)	0.1 (0.1)	0.1 (0.1)
DHA, mean (SD) (g/d)	0.1 (0.1)	0.1 (0.1)
Omega-6 PUFAs, mean (SD) (g/d)	12.4 (4.8)	15.9 (6.1)
Omega-6 to omega-3 PUFA ratio	7.2 (1.8)	7.6 (1.7)
Outcome characteristics		
Time to pregnancy, median (IQR) (mo)	4.8 (1.2–16.3)	—
Time to pregnancy ≥12 mo or use of ART (subfertility), % (*n*)	30.5 (253)	—
Pregnancy results of infertility treatment, % (*n*)	13.2 (108)	—

Abbreviations: ALA, α-linolenic acid, ART, assisted reproductive technology; CI, confidence interval; DHA, Docosahexaenoic acid; EPA, Eicosapentaenoic acid; PUFA, Poly-unsaturated fatty acids.

1 Dutch dietary recommendations of consuming ≥2 servings of seafood per week and ≥25 g of nuts and seeds daily.

Nonresponse analyses revealed that females and males with dietary intake data available were more likely to be of Dutch nationality, highly educated, nulliparous (in females), nonsmokers, alcohol users, and exhibited a lower (prepregnancy) BMI, compared with those without dietary intake data (Supplemental Table 1).

### Periconception PUFA-rich food intake, time to pregnancy, and subfertility

**FIGURE 1:**
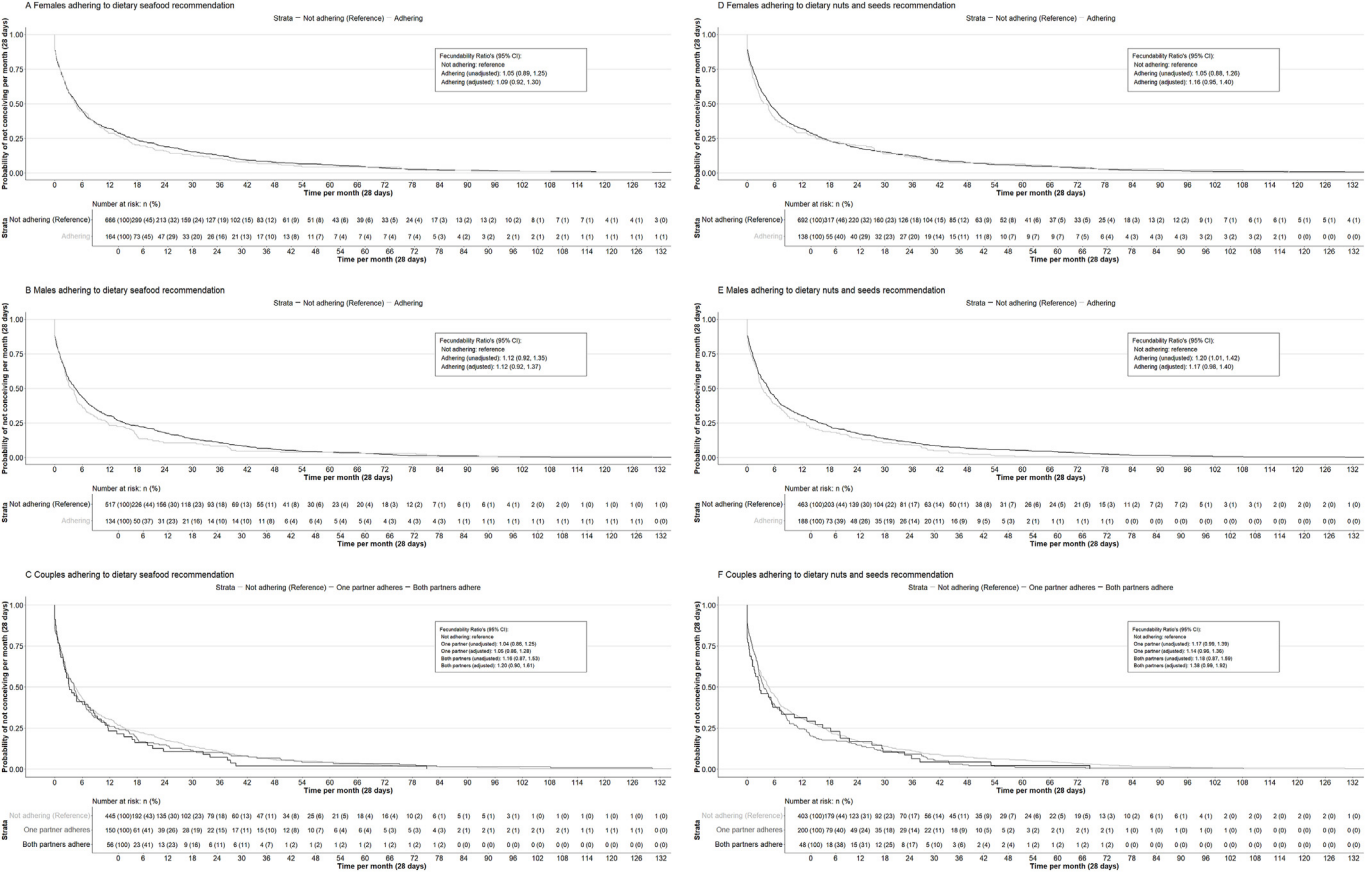
Kaplan–Meier survival curves for females and males adhering to dietary recommendations for seafood (≥2 servings of seafood per week) and nuts and seeds (≥25 g of nuts and seeds daily). The survival curves represent the survival probability [probability of not conceiving per month (28 d)] for (A) females adhering to the dietary seafood recommendation as compared with females not adhering, (B) males adhering to the dietary seafood recommendation as compared with males not adhering, (C) couples adhering to the dietary seafood recommendation as compared with couples not adhering to the dietary recommendation, (D) females adhering to the dietary nuts and seeds recommendation as compared with females not adhering, (E) males adhering to the dietary nuts and seeds recommendation as compared with males not adhering, (F) couples adhering to the dietary nuts and seeds recommendation as compared with couples not adhering to the dietary recommendation. Fecundability ratios (FRs) are shown within the figures and represent the fecundability when compared with the reference category. Fecundability represents the probability of conceiving within 1 mo (28 d). FRs were derived from the hazard ratios of the Cox proportional hazard models. Survival curves are displayed for unadjusted models. Confounder models in females included: age, ethnicity, educational level, alcohol use, smoking, parity, prepregnancy BMI, and total energy intake. Confounder models in males included: age, ethnicity, educational level, alcohol use, smoking, BMI, and total energy intake. Combined confounder models included all confounders listed for both females and males. BMI, body mass index; CI, confidence interval.

**FIGURE 2:**
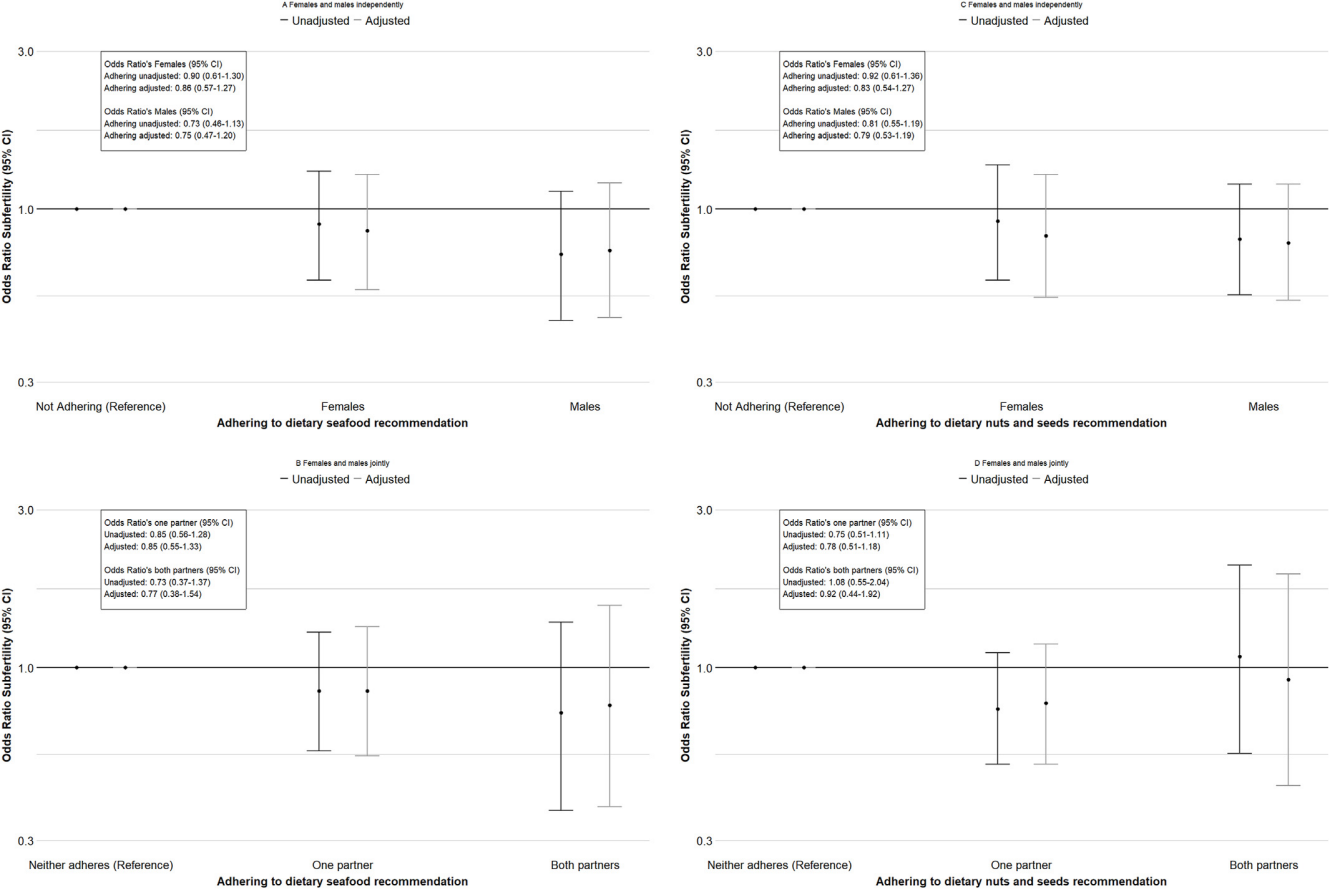
Associations of adhering to dietary recommendations for seafood (≥2 servings of seafood per week) and nuts and seeds (≥25 g of nuts daily) in females and males with odds ratios (OR) of subfertility. Values represent unadjusted and adjusted OR’s of subfertility (time to pregnancy ≥12 mo or use of assisted reproductive technology) as compared with the reference category (not adhering to the dietary recommendation) in (A) females and males independently, and (B) for females and males jointly for the dietary seafood recommendation; and for (C) females and males independently, and (D) for females and males jointly for the dietary nuts and seeds recommendation. Data analyzed using logistic regression models. An OR > 1 indicates increased odds of subfertility compared with the reference category. Confounder models in females included: age, ethnicity, educational level, alcohol use, smoking, parity, prepregnancy BMI, and total energy intake. Confounder models in males included: age, ethnicity, educational level, alcohol use, smoking, parity, BMI, and total energy intake. Combined confounder models included all confounders listed for both females and males. BMI, body mass index; CI, confidence interval.

In females and males, dietary intake of seafood across the full range and seafood consumption frequency were not significantly associated with fecundability and subfertility risk in separate models (Table 2). However, a consistent nonsignificant trend was observed, with increased seafood consumption associated with higher fecundability and lower subfertility risk in females and males, across the full range and in categorized analyses. These tendencies persisted in the combined models, considering both dietary intake of seafood of females and males in 1 model (Table 2). Supplemental Table 2 shows similar tendencies in unadjusted models. In females and males, adherence to dietary seafood recommendations by them individually or jointly was not significantly associated with fecundability or subfertility risk, although a nonsignificant trend toward an association with higher fecundability and a lower subfertility risk with both partners adhering to the dietary seafood recommendation, as compared with neither partner adhering, was present (FIGURE 1, FIGURE 2).

**TABLE 2:** Associations of periconception seafood food intake with fecundability and subfertility risk in females and males.

		Fecundability ratio^1^ (95% CI)		Subfertility Odds ratio^2^ (95% CI)	
		Separate models	Combined models	Separate models	Combined models
Females	*n*				
Seafood consumption (SDS)	830	1.04 (0.97, 1.10)	1.03 (0.95, 1.11)	0.91 (0.78, 1.07)	0.93 (0.77, 1.13)
Seafood consumption frequency					
<1 d/wk	407	Ref	Ref	Ref	Ref
1 to <2 d/wk	259	1.13 (0.97, 1.33)	1.10 (0.91, 1.32)	0.86 (0.60, 1.24)	0.94 (0.61, 1.44)
≥2 d/wk	164	1.15 (0.95, 1.38)	1.17 (0.92, 1.47)	0.80 (0.52, 1.23)	0.87 (0.51, 1.48)
Males					
Seafood consumption (SDS)	651	1.03 (0.96, 1.11)	1.02 (0.95, 1.11)	0.97 (0.80, 1.17)	1.00 (0.82, 1.23)
Seafood consumption frequency					
<1 d/wk	278	Ref	Ref	Ref	Ref
1 to <2 d/wk	239	1.08 (0.90, 1.29)	1.07 (0.89, 1.29)	0.87 (0.58, 1.28)	0.89 (0.58, 1.37)
≥2 d/wk	134	1.16 (0.94, 1.45)	1.10 (0.88, 1.38)	0.71 (0.43, 1.16)	0.75 (0.44, 1.28)

Confounder models in females included: age, ethnicity, educational level, alcohol use, smoking, parity, prepregnancy BMI, and total energy intake.

Confounder models in males included: age, ethnicity, educational level, alcohol use, smoking, BMI, and total energy intake.

Combined confounder models included all confounders listed for both females and males.

Abbreviations: BMI, body mass index; CI, confidence interval; SDS, SD score.

1 Values represent the fecundability per SDS increase in dietary intake of seafood or as compared with the reference category in females and males. Fecundability represents the probability of conceiving within 1 mo (28 d). Models were analyzed using Cox proportional hazard models. Fecundability ratios (FRs) were derived from the hazard ratios of the Cox proportional hazard models.

2 Values represent odds on subfertility (time to pregnancy ≥12 mo or use of assisted reproductive technology) per SDS increase in dietary intake of seafood or as compared with the reference category in females and males. Data analyzed using logistic regression models. Combined models consider the dietary intake of seafood of females and males together in 1 model.

In females, dietary intake of nuts and seeds across the full range and in consumption categories was not significantly associated with fecundability or subfertility risk in separate models (Table 3). No consistent tendencies were present. No associations were present in unadjusted models (Supplemental Table 3). In the combined models, higher dietary intake of nuts and seeds, across the full range, was associated with increased subfertility risk in females. However, no consistent pattern across categorized amounts and no significant associations with fecundability in females were present. In males, higher dietary intake of total nuts and seeds across the full range tended to be associated with higher fecundability in separate models [FR: 1.08, 95% confidence interval (CI): 0.99, 1.17, per SDS increase in total and nuts seeds intake] (Table 3). Strongest effects were present for those who consumed the highest amount (FR: 1.19, 95% CI: 0.98, 1.44, for males in the highest consumption category as compared with the lowest consumption category). In combined models, considering both nuts and seeds intake of females and males, these effects strengthened in males (FR: 1.10, 95% CI: 1.01, 1.20; FR: 1.27, 95% CI: 1.05, 1.54, per SDS increase in total nuts and seeds intake, and for males in the highest consumption category as compared with the lowest consumption category, respectively). Similarly, a higher dietary intake of total nuts and seeds across the full range tended to be associated with decreased subfertility risk in males in the separate models (OR: 0.82, 95% CI: 0.67, 1.01, per SDS increase in total nuts and seeds intake), with the strongest effect for those who consumed the highest amount (OR: 0.67, 95% CI: 0.43, 1.05 for males in the highest consumption category as compared with the lowest category). These effects strengthened in the combined models in males (OR: 0.78, 95% CI: 0.63, 0.97; OR: 0.62, 95% CI: 0.39, 1.00, per SDS increase in total nuts and seeds intake, and for males in the highest consumption category as compared with the lowest consumption category, respectively). Strongest effects in males tended to be present for peanut products. Similar directions of effects were present in unadjusted models (Supplemental Table 3). Adherence to the Dutch dietary nuts and seeds recommendation in males tended to be associated with increased fecundability (FR: 1.17, 95% CI: 0.98, 1.40, as compared with not adhering to the dietary nuts and seeds recommendation), but not with subfertility risk or in females (FIGURE 1, FIGURE 2) (Supplemental Tables 4 and 5). Both partners adhering to the dietary nuts and seeds recommendation tended to be associated with increased fecundability, as compared with neither partner adhering (FR: 1.38, 95% CI: 0.99, 1.92, as compared with neither partner adhering to the nuts and seeds recommendation), but not with subfertility risk (FIGURE 1, FIGURE 2). When repeating the analyses for females and males living with overweight or obesity, we observed largely similar effect estimates (Supplemental Tables 6 and 7).

**TABLE 3:** Associations of periconception nuts and seeds intake with fecundability and subfertility risk in females and males.

	*n*	Fecundability ratio^1^ (95% CI)		Subfertility Odds ratio^2^ (95% CI)	
		Separate models	Combined models	Separate models	Combined models
Females					
Total nuts/seeds (SDS)	830	1.00 (0.93, 1.08)	0.97 (0.89, 1.06)	1.08 (0.92, 1.27)	1.21 (1.00, 1.47)
Peanut products (SDS)	830	1.01 (0.95, 1.09)	0.99 (0.92, 1.08)	0.98 (0.84, 1.15)	1.01 (0.84, 1.23)
Other nuts/ seeds (SDS)	830	0.99 (0.93, 1.06)	0.97 (0.89, 1.05)	1.11 (0.95, 1.30)	1.25 (1.03, 1.51)
Total nuts/seeds consumption categories					
Low	408	Ref	Ref	Ref	Ref
Moderate	221	0.97 (0.81, 1.15)	0.81 (0.66, 0.98)	1.17 (0.80, 1.70)	1.52 (0.97, 2.37)
High	201	1.11 (0.92, 1.33)	0.95 (0.76, 1.18)	1.02 (0.68, 1.53)	1.41 (0.86, 2.32)
Males					
Total nuts/seeds (SDS)	651	1.08 (0.99, 1.17)	1.10 (1.01, 1.20)	0.82 (0.67, 1.01)	0.78 (0.63, 0.97)
Peanut products (SDS)	651	1.06 (0.98, 1.15)	1.08 (0.99, 1.17)	0.81 (0.65, 0.99)	0.79 (0.64, 0.99)
Other nuts/seeds (SDS)	651	1.05 (0.97, 1.14)	1.07 (0.98, 1.16)	0.94 (0.78, 1.13)	0.88 (0.71, 1.09)
Total nuts/seeds consumption categories					
Low	242	Ref	Ref	Ref	Ref
Moderate	176	1.05 (0.86, 1.28)	1.13 (0.92, 1.38)	0.99 (0.64, 1.54)	1.01 (0.63, 1.60)
High	233	1.19 (0.98, 1.44)	1.27 (1.05, 1.54)	0.67 (0.43, 1.05)	0.62 (0.39, 1.00)

Confounder models in females included: age, ethnicity, educational level, alcohol use, smoking, parity, prepregnancy BMI, and total energy intake.

Confounder models in males included: age, ethnicity, educational level, alcohol use, smoking, BMI, and total energy intake.

Combined confounder models included all confounders listed for both females and males.

Abbreviation: BMI, body mass index; CI, confidence interval; SDS, SD score.

1 Values represent the fecundability per SDS increase in dietary intake of nuts and seeds or as compared with the reference category in females and males. Fecundability represents the probability of conceiving within 1 mo (28 d). Models were analyzed using Cox proportional hazard models. Fecundability ratios (FRs) were derived from the hazard ratios of the Cox proportional hazard models.

2 Values represent odds of subfertility (time to pregnancy ≥12 mo or use of assisted reproductive technology) per SDS increase in dietary intake of nuts and seeds or as compared with the reference category in females and males. Data analyzed using logistic regression models. Combined models consider the dietary intake of nuts and seeds of females and males together in 1 model.

### Periconception dietary intake of PUFAs, time to pregnancy, and subfertility

In females, higher dietary intake of total omega-3 PUFAs and a lower dietary omega-6 to omega-3 PUFA ratio were, across the full range, associated with increased fecundability and reduced subfertility risk in separate models (FR: 1.17, 95% CI: 1.07, 1.28; OR: 0.80, 95% CI: 0.65, 0.98, per SDS increase in total omega-3 PUFA intake) (FR: 0.94, 95% CI: 0.91, 0.98; OR: 1.10, 95% CI: 1.01, 1.19, per unit increase in omega-6 to omega-3 PUFA ratio) (Table 4). These effects remained present in the combined models. Strongest effects were present for higher dietary DHA and ALA. Similar direction of effects were visible in unadjusted models and in categorized analyses in quartiles, with the strongest effects observed for females in the highest quartile of dietary omega-3 PUFA intake (Supplemental Tables 8 and 9). No associations were present for total omega-6 PUFA intake (Table 4). In males, higher dietary intake of omega-3 and omega-6 PUFAs, but not the ratio, tended to be associated across the full range with increased fecundability and reduced subfertility in separate models, which fully attenuated for omega-3 PUFAs in the combined models (Table 4). When repeating the analyses for females and males living with overweight or obesity, and adjusting for PUFA supplement use before pregnancy, we observed largely similar effect estimates (Supplemental Tables 10 and 11).

**TABLE 4:** Associations of periconception dietary PUFA intake with fecundability and subfertility risk in females and males.

	*n*	Fecundability ratio^1^ (95% CI)		Subfertility Odds ratio^2^ (95% CI)	
		Separate models	Combined models	Separate models	Combined models
Omega-3 PUFAs (SDS)					
Females	830	1.17 (1.07, 1.28)	1.19 (1.08, 1.32)	0.80 (0.65, 0.98)	0.78 (0.60, 1.01)
Males	651	1.08 (0.98, 1.20)	1.03 (0.93, 1.14)	0.90 (0.71, 1.14)	1.01 (0.78, 1.30)
Omega-6 PUFAs (SDS)					
Females	830	1.07 (0.97, 1.19)	1.06 (0.94, 1.19)	0.91 (0.73, 1.13)	0.99 (0.76, 1.29)
Males	651	1.05 (0.94, 1.17)	1.06 (0.95, 1.18)	0.86 (0.66, 1.12)	0.87 (0.66, 1.16)
Omega-6:omega-3 PUFA ratio					
Females	830	0.94 (0.91, 0.98)	0.92 (0.87, 0.96)	1.10 (1.01, 1.19)	1.14 (1.02, 1.26)
Males	651	0.97 (0.93, 1.01)	1.02 (0.97, 1.07)	1.01 (0.91, 1.13)	0.95 (0.85, 1.07)
ALA (SDS)					
Females	830	1.12 (1.02, 1.23)	1.16 (1.05, 1.27)	0.87 (0.71, 1.06)	0.85 (0.67, 1.10)
Males	651	1.08 (0.97, 1.19)	1.03 (0.93, 1.14)	0.87 (0.69, 1.10)	0.94 (0.73, 1.22)
DHA (SDS)					
Females	830	1.08 (1.01, 1.15)	1.06 (0.98, 1.15)	0.84 (0.70, 1.00)	0.83 (0.67, 1.02)
Males	651	1.02 (0.95, 1.10)	1.00 (0.93, 1.09)	1.05 (0.88, 1.26)	1.13 (0.93, 1.37)
EPA (SDS)					
Females	830	1.07 (1.00, 1.13)	1.05 (0.98, 1.13)	0.85 (0.71, 1.02)	0.86 (0.69, 1.05)
Males	651	1.04 (0.96, 1.12)	1.02 (0.95, 1.11)	1.03 (0.85, 1.24)	1.09 (0.89, 1.33)

Confounder models in females included: age, ethnicity, educational level, alcohol use, smoking, parity, prepregnancy BMI, and total energy intake.

Confounder models in males included: age, ethnicity, educational level, alcohol use, smoking, BMI, and total energy intake.

Combined confounder models included all confounders listed for both females and males.

Abbreviations: ALA, α-linolenic acid; BMI, body mass index; CI, confidence interval; DHA, Docosahexaenoic acid; EPA, Eicosapentaenoic acid; PUFA, Poly-unsaturated fatty acids; SDS, SD score.

1 Values represent the fecundability per SDS increase in dietary intake of PUFAs in females and males. Fecundability represents the probability of conceiving within 1 mo (28 d). Models were analyzed using Cox proportional hazard models. Fecundability ratios (FRs) were derived from the hazard ratios of the Cox proportional hazard models.

2 Values represent odds of subfertility (time to pregnancy ≥12 mo or use of assisted reproductive technology) per SDS increase in dietary intake of PUFAs in females and males. Data analyzed using logistic regression models. Combined models consider the dietary intake of PUFAs of females and males together in 1 model.

## Discussion

We observed that higher dietary intake of omega-3 PUFAs and a lower omega-6 to omega-3 PUFA ratio were across the full range associated with increased fecundability and decreased subfertility risk in females, independent of dietary intake of the male partner. Strongest effects were present for DHA and ALA. In females, weaker tendencies in a similar direction were present for PUFA-rich seafood, but not for nuts and seeds. In males, a higher dietary intake of nuts and seeds across the full range was associated with increased fecundability and decreased subfertility risk, independent of the dietary intake of the female partner. No associations of dietary PUFA intake with fertility outcomes were present in males, suggesting that these effects were not driven by dietary PUFA intake.

### Main findings

Dietary PUFA intake in females may support fertility, yet most research has focused on infertile populations, limiting its generalizability to those trying to conceive naturally [32,33]. Among the general population, we observed that higher dietary omega-3 PUFA intake, especially DHA and ALA, was associated with increased fertility in females, independent of the dietary omega-3 PUFA intake of males. Weaker trends in the same direction were present for PUFA-rich seafood, but not for nuts and seeds. Similarly, in 2 prospective cohort studies of females wishing to conceive from Denmark and North America, total seafood intake was not associated with fecundability [34]. When dietary PUFAs were assessed directly, higher omega-3 PUFA intake was linked to increased fecundability in North-American females [11]. They did not observe a clear dose–response relationship, as the association was primarily driven by females in the highest intake quartile. In contrast, we observed a relatively consistent upward trend across quartiles suggesting that moderate dietary omega-3 PUFA intake in our population may already confer some benefits. No association was observed in Danish females, among whom low omega-3 PUFA intake was rare [11]. This suggests that a threshold effect may exist, where an association is detectable only when intake varies across a broader range or is sufficiently low in part of the population. The stronger associations observed with omega-3 PUFAs compared with seafood intake may stem from the presence of pollutants in seafood, such as mercury and polychlorinated biphenyls. These contaminants may counteract the benefits of omega-3 PUFAs, particularly in populations consuming fish species with higher pollutant levels [35]. This highlights the importance of considering both the quality and source of omega-3 PUFAs in dietary recommendations. We observed no association of dietary omega-6 PUFA intake with fecundability in females, similarly as both preconception cohorts [11]. Interestingly, higher intake of nuts and was associated with a slight tendency toward increased subfertility risk in females. Although these findings were not consistent, they may reflect the relatively high omega-6 PUFA content of these foods. Excessive intake of omega 6 PUFAs relative to omega-3 PUFAs may exacerbate inflammation and potentially impair fertility. This hypothesis is supported by our observation that a lower omega-6 to omega-3 PUFA ratio was consistently linked to increased fecundability and reduced subfertility risk, independent of the PUFA ratio of the male partners. Overall, our findings suggest that the potential fertility benefits of PUFA-rich foods in females may be driven primarily by omega-3 PUFAs, particularly DHA and ALA, and the balance between omega-3 and omega-6 PUFAs, rather than these foods as a whole.

In males, PUFAs have gained attention for their potential benefits on sperm parameters, as they are essential for sperm plasma membrane fluidity. A systematic review of 35 studies suggested that diets rich in PUFA-containing foods, such as seafood, may improve semen quality parameters in males, although most studies were conducted in high-risk populations [4]. Similarly, high nut consumption has been associated with improved sperm quality parameters in healthy males [36,37]. However, research has largely focused on sperm quality rather than fecundability. Partially in line with these previous findings, we observed that higher dietary intake of PUFA-rich nuts and seeds, but not seafood, was consistently associated with increased fecundability and decreased subfertility risk in males, independent of their female partner’s dietary intake. Strongest associations were present for peanuts and peanut products and for those who consumed the highest amount. These findings suggest that even modest increases in nuts and seeds consumption may contribute to improved fertility outcomes at the population level, with the strongest effects for males with high consumption. No significant associations were observed between dietary PUFA intake and fertility outcomes in males. Similarly, an internet-based preconception cohort study including couples attempting spontaneous conception showed that males’ dietary intake of omega-3 and omega-6 PUFAs was not strongly associated with fecundability [38]. These findings suggest that the benefits of nuts and seeds on fertility in males may not be solely attributed to PUFAs, but could also involve other bioactive compounds, such as antioxidants, vitamins, and minerals. We were able to separately analyze peanuts and peanut products compared with other nuts and seeds, recognizing their distinct nutritional profiles. Peanuts are particularly high in folate and arginine, both of which have been positively associated with fertility outcomes and may explain the stronger associations we observed for peanuts and peanut products [39,40]. Taken together, we showed that higher nuts and seeds consumption in males is consistently associated with increased fecundability and decreased subfertility risk, whereas dietary PUFA intake alone showed no association. This indicates that the benefits of nut consumption may extend beyond PUFAs, potentially involving other bioactive compounds.

Adherence to the Dutch dietary guidelines for PUFA-rich foods was low in our study population, with only 17%–29% of participants meeting recommendations. This pattern closely aligns with intake in the general Dutch population [41]. Especially in males, but not in females, adherence to the nuts and seeds recommendation was associated with increased fecundability. When both partners adhered to the seafood or nuts and seeds recommendation, we observed a nonsignificant trend toward increased fecundability and decreased subfertility compared with couples where neither partner adhered. This finding aligns with the growing recognition that dietary habits of both partners may synergistically influence reproductive outcomes. The low adherence to dietary recommendations in our population underscores the need for public health initiatives that not only promote healthier dietary behaviors among couples trying to conceive but also support sustained adherence to these guidelines. Beyond evaluating the impact of dietary recommendations on health outcomes, future research should prioritize identifying effective strategies to improve compliance.

The observed associations between omega-3 PUFA intake in females and nuts and seeds intake in males with fertility outcomes may be explained through several pathways. We adjusted for a wide range of sociodemographic, lifestyle, and dietary factors in both females and males, but the associations could partly reflect other lifestyle or dietary behaviors, as individuals with higher PUFA intake may adhere to an overall healthier diet or lifestyle that positively influences fertility. In females, the beneficial effects of omega-3 PUFAs may be attributed to their anti-inflammatory properties, which are essential for maintaining endometrial receptivity, regulating ovulatory processes, and supporting placental development. Conversely, a higher dietary omega-6 to omega-3 ratio promotes proinflammatory pathways, potentially impairing these reproductive pathways through the increased production of arachidonic-acid-derived eicosanoids, which are strongly linked to inflammation and oxidative stress [42]. In males, potential fertility-enhancing effects of nuts and seeds may partly stem from their high selenium content. Selenium is crucial for spermatogenesis, as it reduces oxidative stress in the testes by being incorporated into selenoproteins, which protect spermatozoa from lipid peroxidation [43,44]. Additionally, nuts and seeds are rich in other bioactive compounds, including folate and arginine, which may synergistically contribute to improved fertility outcomes through their roles in DNA synthesis and repair, as well as blood flow and erectile function [39,40]. Future studies should explore the interplay between dietary PUFAs and other bioactive compounds to better understand their roles in modulating oxidative stress and inflammatory pathways, offering deeper insights into the nutritional mechanisms influencing fertility outcomes in females and males.

Although our findings should be considered hypothesis generating, they are important from an etiological perspective, as they provide novel insights into the role of dietary PUFA consumption on fertility outcomes in the general population. Future studies should focus on combining FFQ data with more precise laboratory-based measurements of nutrient biomarkers, particularly for omega-3 and omega-6 fatty acids, as well as specific contaminants like mercury in males and females. This will help improve the accuracy of dietary intake assessments and allow for a more comprehensive understanding of the relationships between diet and fertility outcomes, and their underlying mechanisms.

### Methodological considerations

Nonresponse analysis revealed differences between females and males who completed the FFQ and those who did not respond. The selection toward a relatively healthy Dutch population may have affected the generalizability of our findings and potentially led to an underestimation of associations. The FFQ is widely used in population-based cohorts to assess habitual dietary intake, as it captures long-term consumption patterns and is less burdensome compared with multiple 24-h recalls. FFQs may be less precise in estimating absolute dietary intake, and dietary assessments may be affected by measurement error and reporting bias. The HELIUS FFQ structured food items based on typical consumption moments throughout the day, making it easier for participants to remember and report what they ate, as it mirrors their daily routine. To minimize participant burden and ensure feasibility, food items included in the FFQ were grouped at the highest hierarchical level. As a consequence, we were unable to differentiate between specific fish types, as certain fish types were grouped based on common consumption patterns. This may have influenced our findings, as different fish types vary in their nutrient composition and potential exposure to environmental contaminants. Similarly, although our data processing allowed us to estimate total omega-6 PUFA intake, it did not enable us to distinguish between individual omega-6 fatty acids. Given that dietary intake was assessed in early pregnancy, females may have slightly modified their diet after conception. However, because all participants were in early pregnancy at time of dietary assessment, any changes would likely be comparable across the study population, reducing the potential impact on our findings. Accuracy of time to pregnancy duration may have been affected by the retrospectively answered questionnaires. To address this, time to pregnancy was reconfirmed during the first trimester visit. Although lifestyle factors were explicitly reported for the preconception and early pregnancy periods ensuring a more accurate assessment of potential confounders, residual confounding remains a limitation due to the observational study design. Other dietary factors, such as carbohydrate quality and protein intake, could also be potential confounders in the observed associations. Due to multicollinearity issues, we were unable to include these dietary factors in our analyses, but we did adjust for total energy intake as overall measure of diet.

In conclusion, among couples in the general population, higher dietary omega-3 PUFAs intake and lower omega-6 to omega-3 PUFA ratio were positively associated with fertility in females. In males, a higher dietary intake of nuts and seeds were associated with improved fertility, which may be driven by other bioactive compounds than dietary PUFA intake. These insights underscore the need for further research to refine gender-specific dietary recommendations to optimize fertility outcomes in couples wishing to conceive.

## Authors contributions

The author’s responsibilities were as follows – MCS, RG, VWVJ: designed research; MCS, RG: conducted research, wrote the paper, and primary responsibility for the final content; MCS: analyzed data; VWVJ, ELB, AGMGJM: critically reviewed the manuscript; and all authors: read and approved the final manuscript.

## Data availability

The data that support the findings of this study are not openly available due to reasons of sensitivity and are available from the corresponding author on reasonable request.

## Funding

The Generation R Study is financially supported by the Erasmus Medical Center, Rotterdam, the Erasmus University Rotterdam and the Netherlands Organization for Health Research and Development. RG received funding from the Netherlands Organization for Health Research and Development (NWO, ZonMw VIDI 09150172110034, and NWO, ZonMW, grant number 05430052110007), from the Dutch Diabetes Foundation (Grant no. 2024.28.001), and an European Research Council Starting Grant (ERC-2024-STG-101161004). VWVJ received a grant from the Netherlands Organization for Health Research and Development (NWO, ZonMw 05430052110007) and a European Research Council Consolidator Grant (ERC-2014-CoG-648916). This project has received funding from the European Union’s Horizon 2020 research and innovation program under the ERA-NET Cofund action (no. 727565), European Joint Programming Initiative “A Healthy Diet for a Healthy Life” (JPI HDHL), EndObesity, ZonMW Netherlands (no. 529051026).

## Conflict of interest

The authors report no conflicts of interest.
