# Supplementary material for: Dietary intake of polyunsaturated fatty acids, their food sources and fertility in females and males: a preconception prospective population-based cohort study
Source: Am J Clin Nutr. 2025 Apr 11;121(6):1354–64. doi: 10.1016/j.ajcnut.2025.04.006 (PMC12226746; doi:10.1016/j.ajcnut.2025.04.006)
Supplement: multimedia component 1 [file mmc1.docx]

**Supplementary Materials**

**Dietary intake of poly-unsaturated fatty acids, their food sources and fertility in females and males: a preconception prospective population-based cohort study**

Mireille C. Schipper^1,2^, Vincent W.V. Jaddoe MD PhD^1,2^, Eline L. Bekkers^1^, Annemarie G.M.G.J. Mulders^3^, Romy Gaillard MD PhD^1,2^.

1.The Generation R Study Group, Erasmus MC, University Medical Center, Rotterdam, the Netherlands.

2.Department of Pediatrics, Sophia’s Children’s Hospital, Erasmus MC, University Medical Center, Rotterdam, the Netherlands.

3.Department of Obstetrics and Gynaecology, Erasmus MC, University Medical Center, Rotterdam, the Netherlands.

**Corresponding author**

Romy Gaillard, The Generation R Study Group, Erasmus University Medical Center, PO Box 2040, 3000 CA Rotterdam, the Netherlands ([r.gaillard@erasmusmc.nl)](mailto:r.gaillard@erasmusmc.nl)). Telephone number: 0031 10 704 3405.

**CONTENTS**

**Supplementary Figure S1.** Timeline of the study design illustrating enrollment, follow-up and timing of data collection

**Supplementary Figure S2.** Flowchart of the study population

**Supplementary Figure S3.** Directed Acyclic Graph representing the pathways between dietary poly-unsaturated fatty acid consumption and fertility

**Supplementary Table S1.** Non-response analysis comparing population characteristics of females and males with and without dietary intake data available

**Supplementary Table S2**. Unadjusted associations of periconception seafood intake with fecundability and subfertility risk in females and males

**Supplementary Table S3.** Unadjusted associations of periconception nuts and seed intake with fecundability and subfertility risk in females and males

**Supplementary Table S4.** Unadjusted associations of adhering to the dietary seafood, nuts and seeds recommendations with fecundability and subfertility risk in females and males

**Supplementary Table S5.** Adjusted associations of adhering to the dietary seafood, nuts and seeds recommendations with fecundability and subfertility risk in females and males

**Supplementary Table S6.** Adjusted associations of periconception seafood intake with fecundability and subfertility risk in females and males with overweight or obesity

**Supplementary Table S7.** Adjusted associations of periconception nuts and seeds intake with fecundability and subfertility risk in females and males with overweight or obesity

**Supplementary Table S8.** Unadjusted associations of periconception dietary PUFA intake with fecundability and subfertility risk in females and males

**Supplementary Table S9.** Adjusted associations of periconception dietary PUFA intake in quartiles with fecundability and subfertility risk in females

**Supplementary Table S10.** Adjusted associations of periconception dietary PUFA intake with fecundability and subfertility risk in females and males with overweight or obesity

**Supplementary Table S11.** Associations of periconception dietary PUFA intake with fecundability and subfertility risk in females additionally adjusted for PUFA supplement use

**
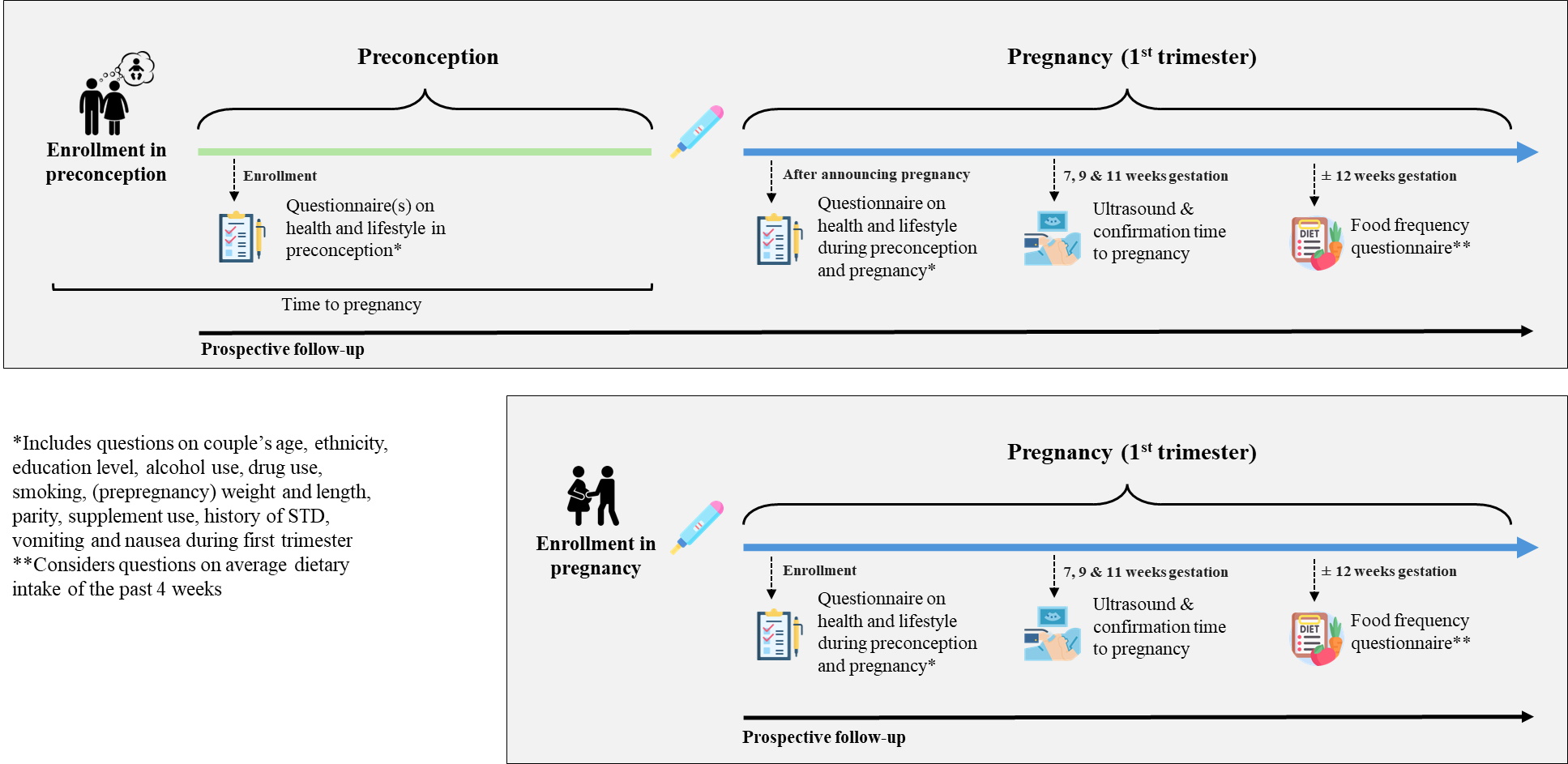
Supplementary Figure S1.** Timeline of the study design illustrating enrollment, follow-up and timing of data collection

**Supplementary Figure S2**. Flowchart of the study population


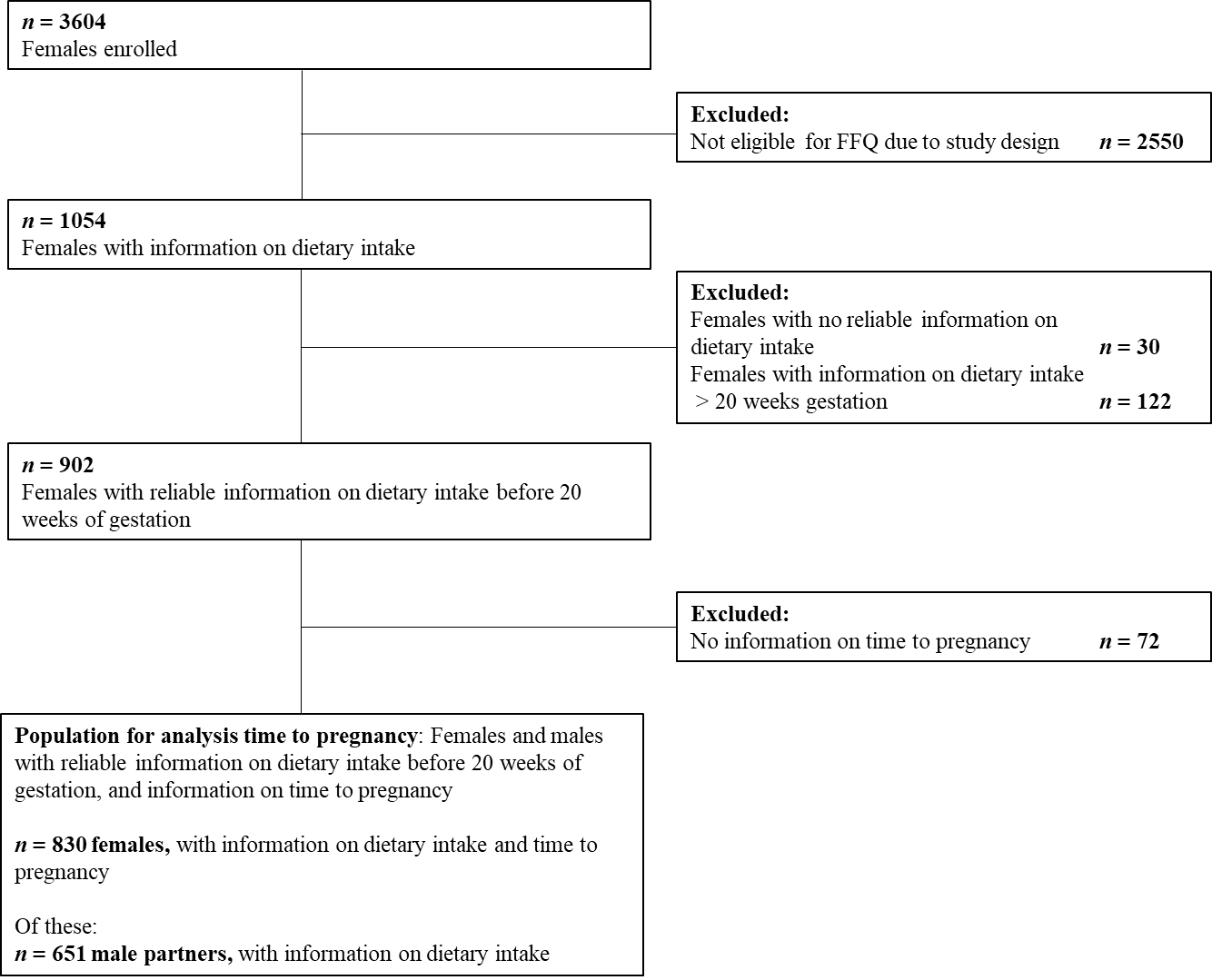


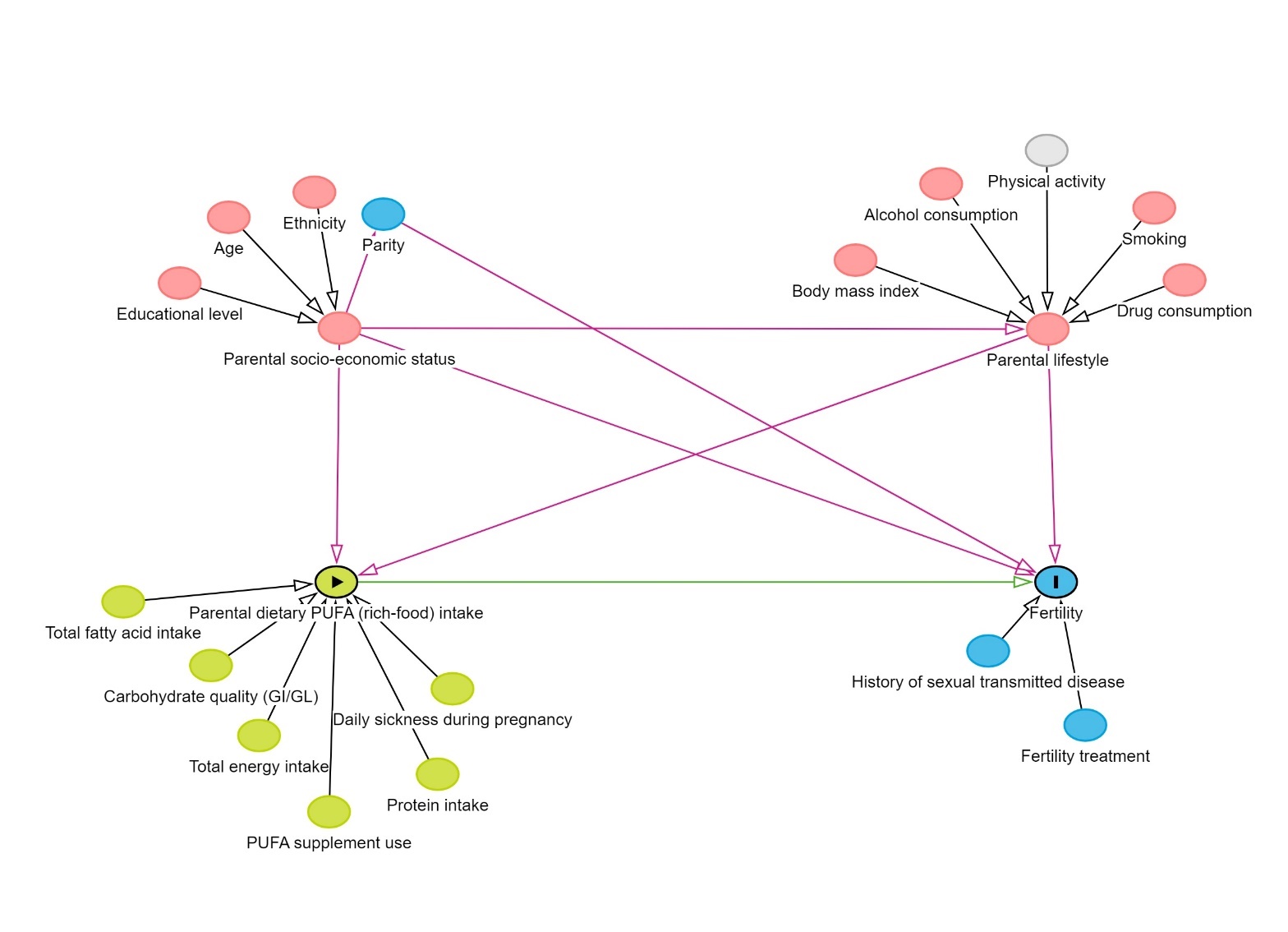
**Supplementary Figure S3.** Directed Acyclic Graph representing the pathways between dietary poly-unsaturated fatty acid consumption and fertility

Directed Acyclic Graph (DAG) representing the assumed causal structure in our study on parental dietary poly-unsaturated fatty acid (PUFA)(-rich food) intake and fertility. Green node indicates our exposure (parental dietary PUFA intake) and blue node (fertility) our outcome. All other green and blue nodes are variables descendent to our exposure and outcome, respectively. Pink nodes indicated potential confounders. The grey nodes are unmeasured confounders. Green lines indicated potential causal pathways, and pink lines biased pathways.

**Supplementary Table S1.** Non-response analysis comparing population characteristics of females and males with and without dietary intake data available

|  | Females without dietary intake data  *n* = 431 | Females with dietary intake data    *n* = 1054 | P-value | Males without dietary intake data  *n* = 595 | Males with dietary intake data  *n* = 902 | P-value |
| --- | --- | --- | --- | --- | --- | --- |
| Ethnicity, % (*n*) |  |  | <0.001 |  |  | <0.001 |
| Dutch | 44.0 (155) | 67.1 (696) |  | 44.0 (209) | 72.8 (647) |  |
| European | 12.8 (45) | 9.7 (101) |  | 10.9 (52) | 8.0 (71) |  |
| Non-European | 43.2 (152) | 23.2 (241) |  | 45.1 (214) | 19.2 (171) |  |
| Education level, high, % (*n*) | 52.4 (183) | 79.4 (819) | <0.001 | 47.5 (225) | 72.2 (644) | <0.001 |
| Parity, nulliparous, % (*n*) | 54.7 (152) | 69.9 (719) | <0.001 | na | na |  |
| Pre-pregnancy body mass index, median (IQR), kg/m^2^ | 24.3 (21.7, 28.3) | 23.1 (21.1, 25.6) | <0.001 | 25.5 (23.2, 28.6) | 24.7 (22.8, 26.9) | <0.001 |
| Overweight/obesity, % (*n*) | 43.5 (114) | 29.2 (290) | <0.001 | 58.2 (210) | 45.7 (408) | <0.001 |
| Periconception folic acid supplement use, % (*n*) | 98.3 (231) | 99.5 (993) | 0.073 | na | na |  |
| Smoking before pregnancy, yes, % (*n*) | 52.6 (131) | 44.0 (403) | 0.016 | 52.9 (198) | 47.9% (421) | 0.11 |
| Alcohol before pregnancy, yes % (*n*) | 70.8 (189) | 82.4 (845) | <0.001 | 82.7 (315) | 90.8 (797) | <0.001 |
| Drug use before pregnancy, yes % (*n*) | 8.2 (26) | 9.8 (102) | 0.4 | na | na |  |
| P-values calculated using *t*-tests, Mann-Whitney *U*-tests, Chi-square tests or Fisher’s Exact tests. | | | | | | |

**Supplementary Table S2.** Unadjusted associations of periconception seafood intake with fecundability and subfertility risk in females and males

|  |  |  | ^1^Fecundability ratio  (95% CI) | Subfertility  ^2^Odds Ratio  (95% CI) |
| --- | --- | --- | --- | --- |
|  |  |  | Unadjusted | |
| Females |  | *n* |  |  |
| Seafood consumption SDS | | 830 | 1.00 (0.95, 1.07) | 0.95 (0.81, 1.10) |
| Seafood consumption frequency | <1 day per week | 407 | *Ref* | *Ref* |
|  | 1 day per week | 259 | 1.08 (0.92, 1.26) | 0.88 (0.63, 1.24) |
|  | ≥2 days per week | 164 | 1.09 (0.90, 1.30) | 0.86 (0.57, 1.27) |
| Males |  |  |  |  |
| Seafood consumption SDS | | 651 | 1.03 (0.96, 1.11) | 0.94 (0.78, 1.12) |
| Seafood consumption frequency | <1 day per week | 278 | *Ref* | *Ref* |
|  | 1 day per week | 239 | 1.07 (0.90, 1.27) | 0.87 (0.59, 1.27) |
|  | ≥2 days per week | 134 | 1.15 (0.94, 1.42) | 0.68 (0.42, 1.09) |
| 1) Values represent the fecundability per SDS increase in dietary intake of seafood or as compared to the reference category, in females and males. Fecundability represents the probability of conceiving within one month (28 days). Models were analyzed using Cox proportional hazard models. Fecundability ratio’s (FRs) were derived from the hazard ratios of the Cox proportional hazards models. 2) Values represent the odds of subfertility (time to pregnancy ≥12 months or use of assisted reproductive technology) per SDS increase in dietary intake of seafood or as compared to the reference category in females and males. Models were analyzed using logistic regression models. | | | | |

**Supplementary Table S3.** Unadjusted associations of periconception nuts and seeds intake with fecundability and subfertility risk in females and males

|  |  |  | ^1^Fecundability ratio  (95% CI) | Subfertility  **^2^**Odds Ratio  (95% CI) |
| --- | --- | --- | --- | --- |
|  |  |  | Unadjusted | |
| Females |  | *n* |  |  |
| Total nuts/seeds consumption (SDS) | | 830 | 0.96 (0.90, 1.03) | 1.10 (0.95, 1.27) |
| Peanut products consumption (SDS) | | 830 | 1.00 (0.93, 1.06) | 1.00 (0.86, 1.16) |
| Other nuts/seeds consumption (SDS) | | 830 | 0.96 (0.90, 1.02) | 1.12 (0.97, 1.30) |
| Total nuts/seeds consumption categories | Low | 408 | *Ref* | *Ref* |
|  | Moderate | 221 | 0.95 (0.81, 1.12) | 1.20 (0.84, 1.70) |
|  | High | 201 | 1.01 (0.85, 1.20) | 1.06 (0.73, 1.53) |
| Males |  |  |  |  |
| Total nuts/seeds consumption (SDS) | | 651 | 1.09 (1.01, 1.17) | 0.84 (0.69, 1.01) |
| Peanut products consumption (SDS) | | 651 | 1.07 (0.99, 1.16) | 0.84 (0.68, 1.01) |
| Other nuts/seeds consumption (SDS) | | 651 | 1.06 (0.98, 1.14) | 0.92 (0.77, 1.10) |
| Total nuts/seeds consumption categories | Low | 242 | *Ref* | *Ref* |
|  | Moderate | 176 | 1.01 (0.83, 1.22) | 1.02 (0.67, 1.56) |
|  | High | 233 | 1.19 (1.00, 1.43) | 0.72 (0.47, 1.07) |
| 1) Values represent the fecundability per SDS increase in dietary intake of nuts and seeds or as compared to the reference category in females and males. Fecundability represents the probability of conceiving within one month (28 days). Models were analyzed using Cox proportional hazard models. Fecundability ratios (FRs) were derived from the hazard ratios of the Cox proportional hazards models. 2) Values represent the odds of subfertility (time to pregnancy ≥12 months or use of assisted reproductive technology) per SDS increase in dietary intake of nuts and seeds or as compared to the reference category in females and males. Models were analyzed using logistic regression models. | | | | |

**Supplementary Table S4.** Unadjusted associations of adhering to the dietary seafood, nuts and seeds recommendations with fecundability and subfertility risk in females and males

|  |  |  | **^1^**Fecundability ratio  (95% CI) | Subfertility  **^2^**Odds Ratio  (95% CI) |
| --- | --- | --- | --- | --- |
|  |  |  | Unadjusted | |
| Adherence to recommendation of ≥2 seafood servings per week | Females | *n* |  |  |
|  | Not adhering | 666 | *Ref* | *Ref* |
|  | Adhering | 164 | 1.05 (0.89, 1.25) | 0.90 (0.61, 1.30) |
|  | Males |  |  |  |
|  | Not adhering | 517 | *Ref* | *Ref* |
|  | Adhering | 134 | 1.12 (0.92, 1.35) | 0.73 (0.46, 1.13) |
|  | Combined |  |  |  |
|  | Neither partner adheres | 445 | *Ref* | *Ref* |
|  | One partner adheres | 150 | 1.04 (0.86, 1.25) | 0.85 (0.56, 1.28) |
|  | Both partners adhere | 56 | 1.16 (0.87, 1.53) | 0.73 (0.37, 1.37) |
| Adherence to recommendation of ≥25 grams of nuts and seeds daily | Females |  |  |  |
|  | Not adhering | 692 | *Ref* | *Ref* |
|  | Adhering | 138 | 1.05 (0.88, 1.26) | 0.92 (0.61, 1.36) |
|  | Males |  |  |  |
|  | Not adhering | 463 | *Ref* | *Ref* |
|  | Adhering | 188 | 1.20 (1.01, 1.42) | 0.81 (0.55, 1.19) |
|  | Combined |  |  |  |
|  | Neither partner adheres | 403 | *Ref* | *Ref* |
|  | One partner adheres | 200 | 1.17 (0.99, 1.39) | 0.75 (0.51, 1.11) |
|  | Both partners adhere | 48 | 1.18 (0.87, 1.59) | 1.08 (0.55, 2.04) |
| 1) Values represent the fecundability as compared to the reference category, in females and males. Fecundability represents the probability of conceiving within one month (28 days). Models were analyzed using Cox proportional hazard models. Fecundability ratios (FRs) were derived from the hazard ratios of the Cox proportional hazards models. 2) Values represent the odds of subfertility (time to pregnancy ≥12 months or use of assisted reproductive technology) as compared to the reference category, in females and males. Models were analyzed using logistic regression models. | | | | |

**Supplementary Table S5.** Adjusted associations of adhering to the dietary seafood, nuts and seeds recommendations with fecundability and subfertility risk in females and males

|  |  |  | ^1^Fecundability ratio  (95% CI) | Subfertility  **^2^**Odds Ratio  (95% CI) |
| --- | --- | --- | --- | --- |
| Adherence to recommendation of ≥2 seafood servings per week | Females | *n* |  |  |
|  | Not adhering | 666 | *Ref* | *Ref* |
|  | Adhering | 164 | 1.09 (0.92, 1.30) | 0.86 (0.57, 1.27) |
|  | Males |  |  |  |
|  | Not adhering | 517 | *Ref* | *Ref* |
|  | Adhering | 134 | 1.12 (0.92, 1.37) | 0.75 (0.47, 1.20) |
|  | Combined |  |  |  |
|  | Neither partner adheres | 445 | *Ref* | *Ref* |
|  | One partner adheres | 150 | 1.05 (0.86, 1.28) | 0.85 (0.54, 1.33) |
|  | Both partners adhere | 56 | 1.20 (0.90, 1.61) | 0.77 (0.38, 1.54) |
| Adherence to recommendation of ≥25 grams of nuts and seeds daily | Females |  |  |  |
|  | Not adhering | 692 | *Ref* | *Ref* |
|  | Adhering | 138 | 1.16 (0.95, 1.40) | 0.83 (0.54, 1.27) |
|  | Males |  |  |  |
|  | Not adhering | 463 | *Ref* | *Ref* |
|  | Adhering | 188 | 1.17 (0.98, 1.40) | 0.79 (0.53, 1.19) |
|  | Combined |  |  |  |
|  | Neither partner adheres | 403 | *Ref* | *Ref* |
|  | One partner adheres | 200 | 1.14 (0.96, 1.36) | 0.78 (0.51, 1.18) |
|  | Both partners adhere | 48 | 1.38 (0.99, 1.92) | 0.92 (0.44, 1.92) |
| 1) Values represent the fecundability as compared to the reference category, in females and males. Fecundability represents the probability of conceiving within one month (28 days). Models were analyzed using Cox proportional hazard models. Fecundability ratios (FRs) were derived from the Hazard Ratios of the Cox proportional hazard models. 2) Values represent odds on subfertility (time to pregnancy ≥12 months or use of assisted reproductive technology) as compared to the reference category, in females and males. Data analyzed using logistic regression models.  Confounder models in females included: age, ethnicity, educational level, alcohol use, smoking, parity, pre-pregnancy body mass index, and total energy intake.  Confounder models in males included: age, ethnicity, educational level, alcohol use, smoking, body mass index, and total energy intake.  Combined confounder models included all confounders listed for both females and males. | | | | |

**Supplementary Table S6.** Adjusted associations of periconception seafood intake with fecundability and subfertility risk in females and males with overweight or obesity

|  |  |  | ^1^Fecundability ratio  (95% CI) | Subfertility  ^2^Odds Ratio  (95% CI) |
| --- | --- | --- | --- | --- |
|  |  |  | Separate models | |
| Females |  | *n* |  |  |
| Seafood consumption (SDS) | | 222 | 1.01 (0.87, 1.18) | 0.89 (0.64, 1.24) |
| Males |  |  |  |  |
| Seafood consumption (SDS) | | 286 | 0.98 (0.87, 1.11) | 1.05 (0.80, 1.39) |
| 1) Values represent the fecundability per SDS increase in dietary intake of seafood in females and males with overweight or obesity. Fecundability represents the probability of conceiving within one month (28 days). Models were analyzed using Cox proportional hazard models. Fecundability ratios (FRs) were derived from the hazard ratios of the Cox proportional hazards models. 2) Values represent the odds of subfertility (time to pregnancy ≥12 months or use of assisted reproductive technology) per SDS increase in dietary intake of seafood in females and males with overweight or obesity. Models were analyzed using logistic regression models.  Confounder models in females included: age, ethnicity, educational level, alcohol use, parity, smoking, and total energy intake.  Confounder models in males included: age, ethnicity, educational level, alcohol use, smoking, and total energy intake.  Combined confounder models included all confounders listed for both females and males. | | | | |

**Supplementary Table S7.** Adjusted associations of periconception nuts and seeds intake with fecundability and subfertility risk in females and males with overweight or obesity

|  |  |  | ^1^Fecundability ratio  (95% CI) | Subfertility  ^2^Odds Ratio  (95% CI) |
| --- | --- | --- | --- | --- |
|  |  |  | Separate models | |
| Females |  | *n* |  |  |
| Total nuts/seeds consumption (SDS) | | 222 | 1.00 (0.85, 1.18) | 1.11 (0.78, 1.58) |
| Peanut products consumption (SDS) | | 222 | 1.02 (0.89, 1.17) | 0.91 (0.68, 1.24) |
| Other nuts/seeds consumption (SDS) | | 222 | 0.97 (0.80, 1.18) | 1.33 (0.88, 2.00) |
| Males |  |  |  |  |
| Total nuts/seeds consumption (SDS) | | 286 | 1.11 (0..97, 1.27) | 0.86 (0.64, 1.17) |
| Peanut products consumption (SDS) | | 286 | 1.11 (0.97, 1.28) | 0.73 (0.52, 1.02) |
| Other nuts/seeds consumption (SDS) | | 286 | 1.05 (0.92, 1.20) | 1.10 (0.82, 1.47) |
| 1) Values represent the fecundability per SDS increase in dietary intake of nuts and seeds in females and males with overweight or obesity. Fecundability represents the probability of conceiving within one month (28 days). Models were analyzed using Cox proportional hazard models. Fecundability ratios (FRs) were derived from the hazard ratios of the Cox proportional hazards models. 2) Values represent the odds of subfertility (time to pregnancy ≥12 months or use of assisted reproductive technology) per SDS increase in dietary intake of nuts and seeds in females and males with overweight or obesity. Models were analyzed using logistic regression models.  Confounder models in females included: age, ethnicity, educational level, alcohol use, smoking, parity, and total energy intake.  Confounder models in males included: age, ethnicity, educational level, alcohol use, smoking, and total energy intake.  Combined confounder models included all confounders listed for both females and males. | | | | |

**Supplementary Table S8.** Unadjusted associations of periconception dietary PUFA intake with fecundability and subfertility risk in females and males

|  |  | | ^1^Fecundability ratio  (95% CI) | Subfertility  **^2^**Odds Ratio  (95% CI) |
| --- | --- | --- | --- | --- |
|  |  | |  |  |
|  |  | *n* | Unadjusted | |
| Omega-3 PUFAs (SDS) | Females | 830 | 1.04 (0.96, 1.12) | 0.95 (0.81, 1.10) |
|  | Males | 651 | 1.07 (0.99, 1.16) | 0.90 (0.75, 1.07) |
| Omega-6 PUFAs (SDS) | Females | 830 | 0.98 (0.92, 1.06) | 1.04 (0.90, 1.20) |
|  | Males | 651 | 1.05 (0.98, 1.13) | 0.90 (0.75, 1.07) |
| Omega-6:omega-3 PUFA ratio | Females | 830 | 0.96 (0.92, 1.00) | 1.08 (1.00, 1.17) |
|  | Males | 651 | 0.98 (0.93, 1.02) | 1.02 (0.92, 1.13) |
| ALA (SDS) | Females | 830 | 1.02 (0.94, 1.10) | 0.98 (0.84, 1.14) |
|  | Males | 651 | 1.07 (0.99, 1.16) | 0.88 (0.73, 1.05) |
| DHA (SDS) | Females | 830 | 1.03 (0.97, 1.10) | 0.88 (0.74, 1.03) |
|  | Males | 651 | 1.03 (0.96, 1.10) | 1.01 (0.85, 1.20) |
| EPA (SDS) | Females | 830 | 1.02 (0.96, 1.08) | 0.90 (0.75, 1.05) |
|  | Males | 651 | 1.04 (0.97, 1.11) | 0.99 (0.82, 1.17) |
| 1) Values represent the fecundability per SDS increase in dietary intake of poly-unsaturated fatty acid (PUFA) in females and males. Fecundability represents the probability of conceiving within one month (28 days). Models were analyzed using Cox proportional hazard models. Fecundability ratios (FRs) were derived from the Hazard Ratios of the Cox proportional hazard models. 2) Values represent odds on subfertility (time to pregnancy ≥12 months or use of assisted reproductive technology) per SDS increase in dietary intake of PUFAs in females and males. Data analyzed using logistic regression models.  Abbreviations: ALA: Alpha-linolenic acid, DHA: Docosahexaenoic acid, EPA: Eicosapentaenoic acid, PUFA: Poly-unsaturated fatty acids | | | | |

**Supplementary Table S9.** Adjusted associations of periconception dietary PUFA intake in quartiles with fecundability and subfertility risk in females

|  |  | ^1^Fecundability ratio  (95% CI) | Subfertility  **^2^**Odds Ratio  (95% CI) |
| --- | --- | --- | --- |
|  | *n* = 830 | Separate models | |
| Omega-3 PUFAs | Quartile 1 | *Ref* | *Ref* |
|  | Quartile 2 | 1.19 (0. 97, 1.45) | 0.66 ( 0.42, 1.04) |
|  | Quartile 3 | 1.11 (0.89, 1.37) | 0.69 (0.43, 1.13) |
|  | Quartile 4 | 1.55 (1.23, 1.97) | 0.39 (0.22, 0.68) |
| Omega-6 PUFAs | Quartile 1 | *Ref* | *Ref* |
|  | Quartile 2 | 0.83 (0.68, 1.02) | 1.75 (1.11, 2.77) |
|  | Quartile 3 | 0.93 (0.75, 1.16) | 1.14 (0.69, 1.89) |
|  | Quartile 4 | 0.96 (0.75, 1.24) | 1.06 (0.59, 1.89) |
| 1) Values represent the fecundability per quartile increase in dietary intake of total omega-3 or omega-6 poly-unsaturated fatty acid (PUFA) in females, as compared to quartile 1. Fecundability represents the probability of conceiving within one month (28 days). Models were analyzed using Cox proportional hazard models. Fecundability ratio’s (FRs) were derived from the Hazard Ratios of the Cox proportional hazard models. 2) Values represent odds of subfertility (time to pregnancy ≥12 months or use of assisted reproductive technology) per quartile increase in dietary intake of total omega-3 and omega-6 PUFAs, as compared to quartile 1, in females. Data analyzed using logistic regression models.  Confounder models included: age, ethnicity, educational level, alcohol use, smoking, parity, pre-pregnancy body mass index, and total energy intake. | | | |

**Supplementary Table S10.** Adjusted associations of periconception dietary PUFA intake with fecundability and subfertility risk in females and males with overweight or obesity

|  |  | | ^1^Fecundability ratio  (95% CI) | Subfertility  ^2^Odds Ratio  (95% CI) |
| --- | --- | --- | --- | --- |
|  |  | *n* | Separate models | |
| Omega-3 PUFAs (SDS) | Females | 222 | 1.11 (0.86, 1.42) | 0.82 (0.52, 1.29) |
|  | Males | 286 | 1.13 (0.98, 1.31) | 0.96 (0.68, 1.37) |
| Omega-6 PUFAs (SDS) | Females | 222 | 1.27 (0.96, 1.68) | 0.85 (0.53, 1.37) |
|  | Males | 286 | 1.09 (0.93, 1.28) | 0.90 (0.61, 1.33) |
| Omega-6:omega-3 PUFA ratio | Females | 222 | 1.02 (0.95, 1.09) | 1.00 (0.86, 1.16) |
|  | Males | 286 | 0.95 (0.89, 1.02) | 1.01 (0.86, 1.18) |
| ALA (SDS) | Females | 222 | 1.09 (0.84, 1.41) | 0.95 (0.61, 1.48) |
|  | Males | 286 | 1.15 (0.99, 1.34) | 0.91 (0.64, 1.30) |
| DHA (SDS) | Females | 222 | 1.05 (0.88, 1.25) | 0.73 (0.49, 1.09) |
|  | Males | 286 | 1.01 (0.91, 1.13) | 1.08 (0.82, 1.41) |
| EPA (SDS) | Females | 222 | 1.07 (0.90, 1.28) | 0.75 (0.50, 1.13) |
|  | Males | 286 | 1.01 (0.90, 1.13) | 1.11 (0.84, 1.46) |
| 1) Values represent the fecundability per SDS increase in dietary intake of poly-unsaturated fatty acids (PUFAs) in females and males with overweight or obesity. Fecundability represents the probability of conceiving within one month (28 days). Models were analyzed using Cox proportional hazard models. Fecundability ratios (FRs) were derived from the Hazard Ratios of the Cox proportional hazard models. 2) Values represent odds on subfertility (time to pregnancy ≥12 months or use of assisted reproductive technology) per SDS increase in dietary intake of PUFAs in females and males with overweight or obesity. Data analyzed using logistic regression models.  Confounder models in females included: age, ethnicity, educational level, alcohol use, smoking, parity, and total energy intake.  Confounder models in males included: age, ethnicity, educational level, alcohol use, smoking, and total energy intake.  Combined confounder models included all confounders listed for both females and males.  Abbreviations: ALA: Alpha-linolenic acid, DHA: Docosahexaenoic acid, EPA: Eicosapentaenoic acid, PUFA: Poly-unsaturated fatty acids. | | | | |

**Supplementary Table S11**. Associations of periconception dietary PUFA intake with fecundability and subfertility risk in females additionally adjusted for PUFA supplement use.

|  |  |  | ^1^Fecundability ratio  (95% CI) | Subfertility  ^2^Odds Ratio  (95% CI) |
| --- | --- | --- | --- | --- |
|  |  | *n* | Separate models | |
| Omega-3 PUFAs (SDS) | | 830 | 1.17 (1.06, 1.28) | 0.80 (0.65, 0.98) |
| Omega-6 PUFAs (SDS) | | 830 | 1.07 (0.97, 1.18) | 0.91 (0.74, 1.14) |
| Omega-6:omega-3 PUFA ratio | | 830 | 0.94 (0.91, 0.98) | 1.10 (1.01, 1.20) |
| ALA (SDS) | | 830 | 1.12 (1.02, 1.23) | 0.87 (0.71, 1.06) |
| DHA (SDS) | | 830 | 1.08 (1.01, 1.15) | 0.84 (0.70, 1.00) |
| EPA (SDS) | | 830 | 1.06 (1.00, 1.13) | 0.85 (0.71, 1.01) |
| 1) Values represent the fecundability per SDS increase in dietary intake of poly-unsaturated fatty acids (PUFAs) in females additionally adjusted for PUFA supplement use. Fecundability represents the probability of conceiving within one month (28 days). Models were analyzed using Cox proportional hazard models. Fecundability ratios (FRs) were derived from the Hazard Ratios of the Cox proportional hazard models. 2) Values represent odds on subfertility (time to pregnancy ≥12 months or use of assisted reproductive technology) per SDS increase in dietary intake of PUFAs in females additionally adjusted for PUFA supplement use. Data analyzed using logistic regression models.  Confounder models included: age, ethnicity, educational level, alcohol use, parity, smoking, pre-pregnancy body mass index, total energy intake and PUFA supplement use.  Abbreviations: ALA: Alpha-linolenic acid, DHA: Docosahexaenoic acid, EPA: Eicosapentaenoic acid, PUFA: Poly-unsaturated fatty acids. | | | | |
